# Classification with 2-D convolutional neural networks for breast cancer diagnosis

**DOI:** 10.1038/s41598-022-26378-6

**Published:** 2022-12-17

**Authors:** Anuraganand Sharma, Dinesh Kumar

**Affiliations:** School of Information Technology, Engineering, Maths and Physics, The University of the South Pacific, Suva, Fiji

**Keywords:** Computational biology and bioinformatics, Computer science

## Abstract

Breast cancer is the most common cancer in women. Classification of cancer/non-cancer patients with clinical records requires high sensitivity and specificity for an acceptable diagnosis test. The state-of-the-art classification model—convolutional neural network (CNN), however, cannot be used with such kind of tabular clinical data that are represented in 1-D format. CNN has been designed to work on a set of 2-D matrices whose elements show some correlation with neighboring elements such as in image data. Conversely, the data examples represented as a set of 1-D vectors—apart from the time series data—cannot be used with CNN, but with other classification models such as Recurrent Neural Networks for tabular data or Random Forest. We have proposed three novel preprocessing methods of data wrangling that transform a 1-D data vector, to a 2-D graphical image with appropriate correlations among the fields to be processed on CNN. We tested our methods on Wisconsin Original Breast Cancer (WBC) and Wisconsin Diagnostic Breast Cancer (WDBC) datasets. To our knowledge, this work is novel on non-image tabular data to image data transformation for the non-time series data. The transformed data processed with CNN using VGGnet-16 shows competitive results for the WBC dataset and outperforms other known methods for the WDBC dataset.

## Introduction

In recent times, there are growing interest in the development of machine learning (ML) models for medical datasets due to the advancements in digital technology and improvements in data collection methods. Increasingly, several ML-based systems have been designed as an early warning or diagnostic tool for chronic illnesses, for example diagnosing depression, diabetes and cancer^1^. Breast cancer is arguably one of the deadliest forms of cancer amongst women with millions of reported cases around the world of which many cases become fatal^2,3^. Breast cancer is caused by abnormal growth of some of the breast cells in the lining of the milk glands or ducts of the breast (ductal epithelium)^4,5^. Compared to healthy cells, these cells divide more rapidly and accumulate, forming a lump or mass. At this stage, the cells become malignant and may spread through the breast to lymph nodes or other parts of the body.

The study of breast cancer has attracted considerable attention in the past decades. Improving data collection and storage technologies has resulted in various types and amounts of data collected on breast cancer from around the world. These include data on Ribonucleic Acid (RNA) signatures for cell mutations that cause breast cancer^6,7^, mammogram images^8,9^ and data on symptoms and diagnosis^10^. Many traditional Computer-Aided Diagnosis (CADx) systems require hand-crafted feature extraction which is a challenging task^11,12^. Even conventional ML techniques require the extraction of an optimal set of features manually prior to model training. An extensive review on various feature selection and extraction techniques can be found in^13,14^. Some commonly used approaches for ML models are Principal Component Analysis (PCA)^15^, information gain^16^, GA-based feature selection^17^, recursive feature elimination (RFE)^18^, meta-heuristic methods^19^ and rough sets^20^. Feature selection and extraction, therefore, is an important consideration in the pre-processing step before applying any ML algorithm such as decision trees, Bayesian models, Support Vector Machines (SVM) and Artificial Neural Networks (ANN). The behavior of ML algorithms and their prediction accuracy is influenced by the choice of features selected^21,22^. Many times manual feature extraction or knowledge of domain experts is needed to have a good understanding on the relevance of the attributes^23^.

To address these issues surrounding the use of conventional ML algorithms has propelled the need for new approaches and methods to automatically extract features from large datasets. As a result, Deep Learning (DL) algorithms such as convolutional neural network (CNN or ConvNet) and Recurrent Neural Networks (RNNs) have emerged in recent times that can accept raw data and are automatically able to discover patterns in them^24,25^.

CNN is one of the most popular algorithms for deep learning which is mostly used for image classification, natural language processing, and time series forecasting. Its ability to extract and recognize the fine features has led to the state-of-the-art performance in various application domains such as computer vision, image recognition, speech recognition, natural and language processing^26–28^. CNN is an enhancement of a canonical Neural Networks architecture that is specifically designed for image recognition in^29^. Since then many variations have been added to the architecture of CNN to enhance its ability to produce remarkable solutions for deep learning problems such as AlexNet^26^, VGG Net^27^ and GoogLeNet^30^. CNN eliminates the need for manual feature extraction because the features are learned directly by different convolutional layers^26,31^. It does not require a separate feature extraction strategy which requires domain expert and other preprocessing techniques where complete features may still not be extracted^32^. Despite its huge success with image data, CNN is not designed to handle tabular non-image data in non-time series form. Note that all future referencing of non-image data are in non-time series form unless otherwise specified. Arguably, any problem that can represent the correlation of features of a given data example in a single map, maybe attempted via CNN.

CNNs have proven to work best on data that are in 2-D form, such as images and audio spectrograms^33^. This is attributed to the fact that the convolution technique in CNN requires data examples to have at least two dimensions. Conversely, CNN has been explored on application-specific 1-D data as well. These include gene sequencing data such as DNA sequences being treated as text data (sequence of words)^34^, and signals and sequences in text mining, word detection and natural language processing (NLP)^35,36^. More specifically, CNN for Time-Series Classification (TSC) has been recently explored with some new methods such as Multi-Scale CNN (MCNN)^25^ and an ensemble of CNN models with AlexNet on Inception-v4 architecture^37,38^. These methods have made significant improvement in the accuracy of the classifiers with the state-of-the-art ensemble methods such as Flat-COTE and HIVE-COTE^39,40^. Moreover, raw time-series data has also been used into 1-D CNN by calculating the area of the signal for convolution with better time complexity and scalability^41,42^. Nonetheless, much data still exists in a 1-D format such as clinical data of medical records, and therefore, opens challenging research questions on whether they can be effectively trained for classification using CNN. This paper is aimed at filling this gap by proposing a novel non-time series 1-D numerical data to 2-D data transformation methods and processing them with CNN. This would certainly help machine learners to train their data without being bothered about issues with feature extraction. This can also reduce a large feature vector to just a single image.

This paper is organized as follows: Section “Motivation” demonstrates the theoretical motivation of the proposed method. Section “Proposed methods” describes our three proposed methods of data wrangling from non-image Breast Cancer tabular data^10^ to image data. Section “Experiments” describes the complete methodology of the classification of breast cancer data with CNN. Section “Results” shows the experimental results and Section “Discussion” discusses the outcome of the experiments. Lastly, Section “Conclusion” concludes the paper by summarizing the results and proposing some further extensions to the research.

## Motivation

The main motivation for this paper is to realize the potential of CNN for non-image clinical data for breast cancer because it eliminates the need for manual feature extraction. The features are learned directly by CNN whereby it also produces state-of-the-art recognition results^43^. The key difference between traditional ML and DL is in how features are extracted. Traditional ML approaches use handcrafted engineering features by applying several feature extraction algorithms and then apply the learning algorithms. On the other hand, in the case of DL, the features are learned automatically and are represented hierarchically at multiple levels. This is the strong point of DL against traditional machine learning approaches^43^.

**Figure 1:**
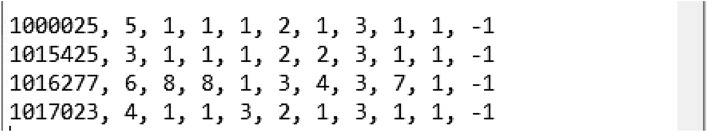
Snapshot of data file for breast cancer dataset WBC from^10^.

We have proposed three novel methods to transform non-image clinical tabular data of breast cancer to 2-D feature map images in $\mathbb {R}^2$ so that a large set of these kinds of data are not deprived of the services of CNN. This would also encourage other variations and/or methods for text to image transformation to be developed in the future. The scope of this paper is to broaden the usage of CNN to those applications where *d*-dimensional raw data has set of *N*, 1-D data vectors in $\mathbb {R}$ as shown in Fig. 1. Each row represents a 1-D data vector with *d* elements where *d*, *N* $\ge 1$. It is a sample of a Wisconsin Original Breast Cancer dataset (WBC) used in the experiments. This dataset from UCI^10^ is a record of medical examination of patients to diagnose breast cancer, where each row is a 1-D vector representing a numerical data example. We demonstrate our method of non-image breast cancer data transformation to image data—processed in CNN—produces exceptional results for classification accuracy. Some research demonstrates the use of 1-D convolutions on 1D datasets such as data in the form of signals and time sequences^44^. Though this provides a possibility of using 1-D convolutions in this research, our experiments revealed their unsuitability on our experimental datasets. Having applied the data in its raw form into 1-D CNN gave highly unpredictable results.

## Proposed methods

We have proposed three basic techniques of data wrangling to convert Breast Cancer numerical tabular data to image data. The converted image must reflect some patterns to depict a given class. We have used Wisconsin Original Breast Cancer (WBC) and Wisconsin Diagnostic Breast Cancer (WDBC) datasets from the UCI library^10^ for the classification of numerical data in this work.

### Equidistant bar graphs

**Figure 2:**
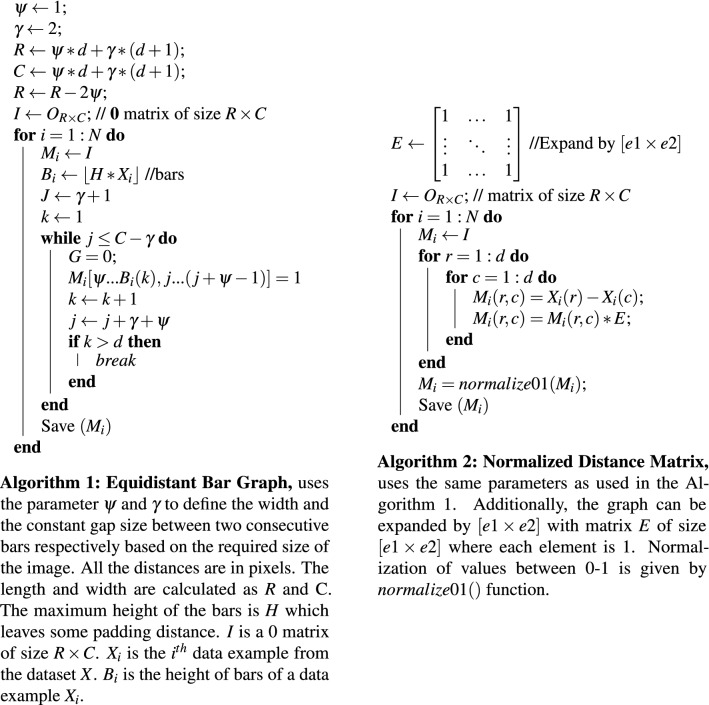
Algorithms for data transformation.

The bar graph represents the measurement of every feature of a given dataset. There are lots of possibilities of drawing a bar graph but we have used a simplistic approach. The dataset is first normalized to [0, 1] then every feature is drawn based on its measured value. The width of the image in pixels is $\psi d+\gamma (d+1)$ where *d* is total features, $\psi$ is the width of a bar and $\gamma$ is gap between two consecutive bars. The height of the image is normalized to produce a square image. We used $1-$pixel length for $\psi$ and $2-$pixels length for $\gamma$ in our experiments. This produces the square image of size $[3d\times 3d]$ approximately. Few data examples of WDBC dataset converted to bar graphs are shown in Fig. 4a with class labels—Benign and Malignant. The algorithm for this approach is given in the Fig. 2 (Algorithm 1).

These pictures are only useful to CNN if they depict a pattern in a convolved image. The first convolutional layer produces 6 features which are shown in Fig. 4b where some sort of distinguishing features have been reflected.

Intuitively, the “correct” order of the bars ought to give better results. The datasets of numerical data were reorganized where the related fields were put close to each other according to the order of their similarity. Firstly, a covariance matrix on data fields was generated then each value of the matrix is converted to ‘rank’ that determines how closely one field is related to the other. This is a shortest-path problem where algorithms such as dynamic programming or any metaheuristic algorithm^45^ such as Genetic Algorithm (GA)^46^, Particle Swarm Optimization^47^ or Reincarnation Algorithm (RA)^48^ can be used to get the optimum order of bars based on their respective rank. Thereafter, a new set of images was created using this new order of bars. This process has been elaborated more in Section “Discussion”.

### Normalized distance matrix

The next method is the formation of a distance matrix which is a squared matrix of size $[d\times d]$ where *d* represents total features of a given example. Matrix elements are the difference between two features i.e., $x_{ij}=x_i-x_j$ where $x_i$ and $x_j$ represent the measurement of a given feature with $i,j\in [1,d]$. We used Euclidean distance in our experiments. The matrix is then normalized between $[0-1]$. This produces the square image of size $[d \times d]$ which has a gain of 3 folds in length compared to bar graphs described in Section “Equidistant bar graphs”. Few data examples of WDBC dataset converted to normalized distance matrix are shown in Fig. 4c with class labels. The images can be easily scaled up to $[3d\times 3d]$. The first convolutional layer produces 6 features similar to bar graphs is shown in Fig. 4d. Its pseudocode and further description is given in Fig. 2 (Algorithm 2).

**Figure 3:**
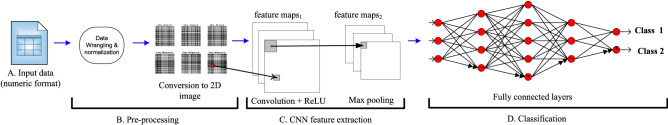
A complete process of non-image tabular data classification with CNN.

**Figure 4:**
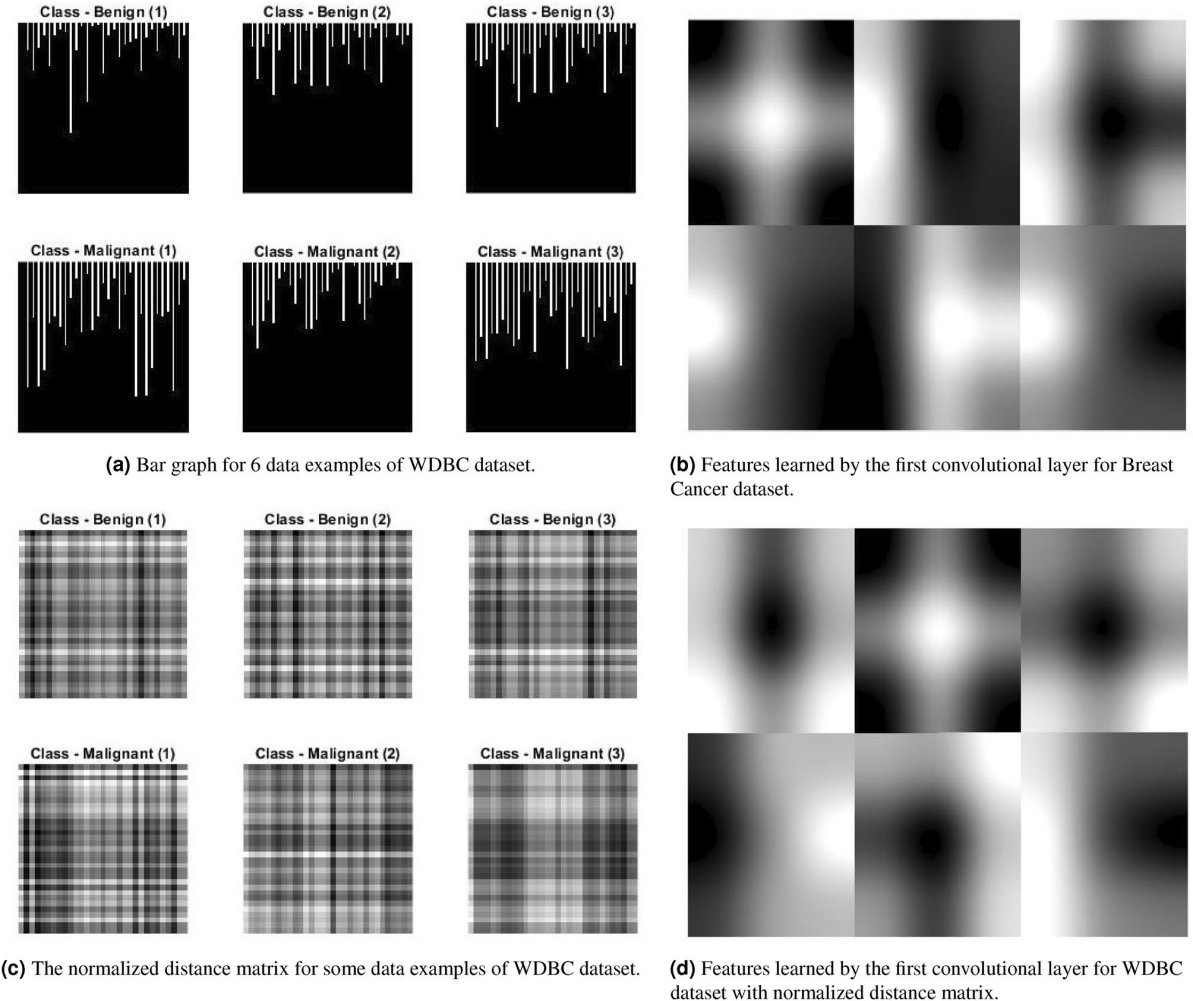
Transformation of tabular data to image and then convolution with CNN.

### Combination of options (bar graph, distance matrix, normalized numeric data)

**Figure 5:**
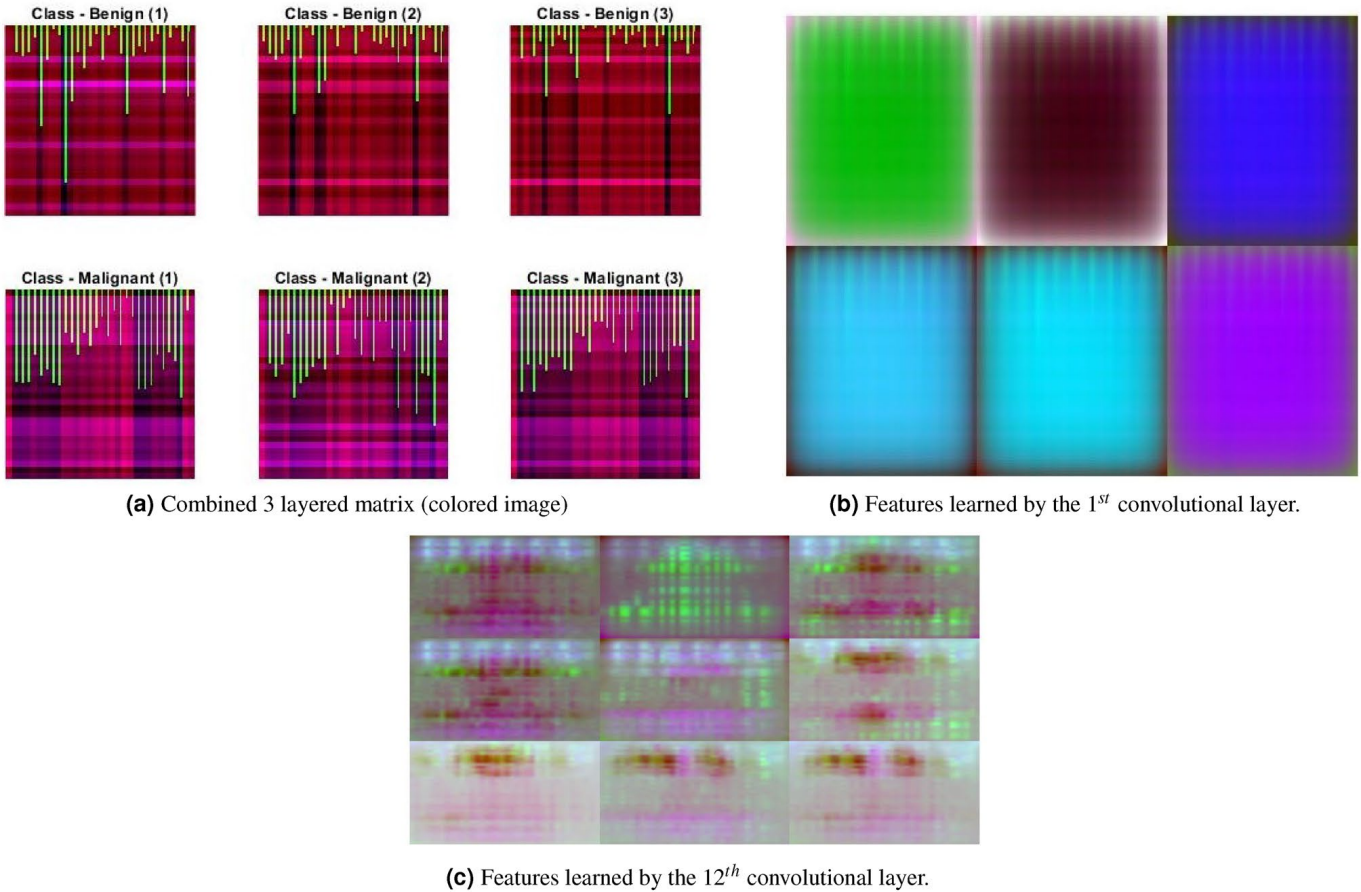
Combined transformation technique and its corresponding convolution with CNN for some data examples of WDBC dataset.

Apparently, the above two strategies can be combined to give a third option for generating an image from numerical data. We create a colored image of 3 layers of size $[3d\times 3d]$ where the first layer has a normalized distance matrix, the second layer has bar graphs, and the third layer has a copy of numerical data stored row-wise, i.e., $x_{ij}=x_i$ where $i,j\in [1,d]$ shows row and column of a matrix and $x_i$ represents the measurement of a given feature. Few data examples of WDBC dataset converted to the combination of options are shown in Fig. 5a with the class labels.

The first convolutional layer in this case, is not able to produce any distinct feature but the scaled up image shows different colors with some bars in Fig. 5b. The 3rd convolved block (12th layer) produces some blobs scattered in the images in Fig. 5c.

**Table 1:** Parameter setting for CNN.

Parameter	Value
Max iterations	1000
Attempts	30
Filter size	3 $\times$ 3
Initial learning rate $\eta$ (with log transformation)	0.02
Momentum	0.88
L2 regularization	9.4E$-$7
Batch size	8

## Experiments

CNN completes the classification process in two steps. The first step is the auto-feature extraction of the images and the second step is the classification of the same images with backpropagation neural networks^49,50^. In the case of a numerical dataset that is not in the form of images, first goes through the data wrangling process described in Section “Proposed methods”, where either of the three options is used for non-image to image data conversion. The transformed images may not make logical sense to human eyes but CNN is capable to extract relevant features out of it. Figure 3 illustrates the complete flowchart of the training process of CNN with non-image data sets. The process contains four important parts: Firstly, numeric input data (A) undergoes pre-processing of data wrangling (B) where it is normalized and converted to 2D image format using one of the data wrangling techniques described in Section “Proposed methods” (the figure shows distance matrix method of Section “Normalized distance matrix”). The generated image is filtered through the CNN convolution layers for feature extraction (C). The features are trained in the fully connected layers to obtain classification outputs (D).

**Table 2:** Experimented dataset.

Dataset	Attributes	Instances	Missing values	Class ratio (Benign:Malignant)
WDBC	32	569	0	357:212
WBC	10	699	16	458:241

## Results

The objective of the experiment is to provide an alternative classification method with CNN for the non-image dataset of Breast Cancer and other similar datasets without any need for manual feature selection. We have used WBC and WDBC datasets from the UCI library^10^ for the experiments. The properties of these datasets are given in Table 2. We have tested the efficacy of our method with other published state-of-the-art methods used for breast cancer diagnosis, namely, variations of Neural Networks (NN)^51^, Support Vector Machine (SVM)^16,52,53^, Decision Tree (DT)^54^ and Naïve Bayes (NB)^55^. These methods are generally supported by additional feature selection methods such as IG, Rough set or weight NB.

For CNN, we used VGG16^27^ architecture with 4 convolutional blocks. Each convolutional block has 2D convolutional layer with the filter size of $[3\times 3]$, $0.5\times Layer\times \left| \root \of {\parallel image \parallel }\right|$ filters, ReLU layer and lastly max pooling layer with of pool size and stride of $[2\times 2]$. Additionally, Bayesian optimization was used for parameter tuning. All parameter settings are shown in Table 1. For regularization and initial learning rate we used log transformation.

Initially, both datasets are divided into $80\%$ training and $20\%$ testing then $20\%$ of training data is kept aside for validation data. After 30 attempts on each dataset, we have collected best and average classification accuracies on validation and test data sets shown in Tables 3 and 4 respectively. Bold figures represent the overall best result. CNN types 1, 2 and 3 represent equidistant bar graph, normalized distant matrix, and combined options respectively. *px*1 shows that the image is formed with bars of 1-pixel width only. Similarly *px*2 and *px*4 show width of 2 and 4 pixel sizes respectively for bars in an image.

**Table 3:** Best results obtained on classification accuracy.

Dataset	Transformation type	Image size					
		px1		px2		px4	
		Val	Test	Val	Test	Val	Test
WDBC	1 - Bar graph	100.00	99.12	98.90	97.35	98.90	98.23
	2 - Dist matrix	98.90	94.69	97.80	96.46	97.80	97.35
	3 - Combined	98.90	**100.00**	98.90	99.12	100.00	98.23
WBC	1 - Bar graph	100.00	98.54	100.00	98.54	100.00	**99.27**
	2 - Dist matrix	100.00	97.08	99.08	**99.27**	98.17	97.81
	3 - Combined	100.00	**99.27**	100.00	98.54	100.00	**99.27**

Significant values are in bold.

**Table 4:** Average results for classification accuracy.

Dataset	Transformation type	Image size					
		px1		px2		px4	
		Val	Test	Val	Test	Val	Test
WDBC	1 - Bar graph	96.37	95.19	96.81	95.25	96.56	95.87
	2 - Dist matrix	93.99	91.21	92.60	91.47	93.19	91.95
	3 - Combined	96.70	**96.02**	96.15	95.01	96.19	95.07
WBC	1 - Bar graph	97.22	95.99	97.71	96.23	97.71	95.55
	2 - Dist matrix	94.25	92.77	94.31	93.67	95.60	94.11
	3 - Combined	97.06	96.40	97.49	**96.55**	97.19	96.08

Significant values are in bold.

Additionally, it is highly desirable in medical diagnosis to have high sensitivity and specificity measures. Sensitivity is the ability of a test to correctly identify those with the disease, and specificity is correctly identifying those without the disease. Alternatively, the F1 score can be used as a derived metric that merges both sensitivity and precision measures. Tables 6 and 7 show the best and average of these additional metrics respectively, for WDBC and WBC datasets on classification. The confusion matrix for the best cases is shown in Table 5. We have also performed experiments using CNN with 1-D convolutions on raw data without any sophisticated data transformation. However, we have obtained poor results when compared to our method with the average classification accuracy of 76.11% and 89.64% for WDBC and WBC datasets respectively.

**Table 5:** Confusion matrices.

	Predicted	
	(0) malignant	(1) benign
**(a) Confusion matrix format**		
**Real**		
(0) malignant	TN	FP
(1) benign	FN	TP
**(b) WDBC (best sensitivity (1.00) & specificity (1.00))**		
**Real**		
(0) malignant	71	0
(1) benign	0	42
**(c) WBC for best sensitivity (1.00)**		
**Real**		
(0) malignant	88	1
(1) benign	0	48
**(d) WBC for best specificity (1.00)**		
**Real**		
(0) malignant	89	0
(1) benign	2	46

**Table 6:** Best score with Type3 on px1.

Dataset	Score type	Score			
		Sensitivity	Specificity	F1	Run time (s)
WDBC	Best sensitivity	1.00	1.00	1.00	13.3
	Best specificity	1.00	1.00	1.00	9.8
WBC	Best sensitivity	1.00	0.99	0.99	15.9
	Best specificity	0.96	1.00	0.98	12.8

The comparison of our methods with other state-of-the-art methods is shown in Table 8. The table shows different methods from 2009–2019. The results show accuracy, sensitivity and specificity of WBC and/or WDBC datasets. Authors in^11^ have used mammogram images of breast cancer as CNN works on images. In some cases, authors got 100% accuracy with 10-fold cross-validation for WBC dataset. Lower fold of cross-validation generally gives lower accuracy^16,51,52^.

**Table 7:** Average score with Type3 on px1.

Dataset	Score type	Avg score	Run time
WDBC	Specificity	0.96	13.2 s
	Sensitivity	0.96	
	F1	0.94	
WBC	Specificity	0.97	13.5 s
	Sensitivity	0.97	
	F1	0.96

**Table 8:** Comparison of the proposed method with other methods.

Authors	Year	Method	Accuracy (%)	Sensitivity	Specificity	Dataset
Akay	2009	SVM with F-score feature selection	99.51	100	97.91	WBC
Chen et al.	2011	Rough set (RS) and SVM	**100**	**100**	**100**	WBC
Onan	2015	Fuzzy-rough nearest neighbor	99.72	100	99.47	WBC
Bhardwaj et al.	2015	Genetically Optimized NN	**100**	98.77	**100**	WBC
Karabatak	2015	Naïve Bayesian (NB)	98.54	99.11	98.25	WBC
Wang et al.	2018	SVM based ensemble learning	97.10	97.11	97.23	WBC
Na Liu et al.	2019	IGSAGAW with CSSVM	95.80	–	–	WBC
Of this paper	2020	CNN with Type-3 Transformation	99.27	**100**	98.88	WBC
Ahn et al.	2009	Novel CBR	99.12	–	–	WDBC
Sun et al.	2017	*CNN on mammogram images*	*82.43*	*81.00*	*72.26*	*Mammogram*
Wang et al.	2018	SVM based ensemble learning	97.68	94.75	99.49	WDBC
Na Liu et al.	2019	IGSAGAW with CSSVM	95.70	–	–	WDBC
Of this paper	2020	CNN with Type-3 Transformation	**100**	**100**	100	WDBC

Significant values are in [bold/italics].

## Discussion

The experimental results of data transformation from non-image tabular breast cancer datasets to image have been promising for the utilization of CNN for classification accuracy. Although the proposed methods are in the early stages, the obtained results are very significant in the development of new strategies with data wrangling for deep learning. This also provides an opportunity to derive even better alternatives for CNN in the future. It was observed that our proposed combined approach, i.e. Type-3 transformation and bar width of 1 pixel i.e. *px*1, has been the most significant method as it carries the most information about the data in three dimensions of an image. It has outperformed other methods for the WDBC dataset by clocking 100% accuracy (with 1.0 sensitivity, specificity and F1 score). It has also shown very competitive results for the WBC dataset with 99.27% accuracy and 1.0 sensitivity 0.99 specificity and 0.99 F1 score.

**Figure 6:**
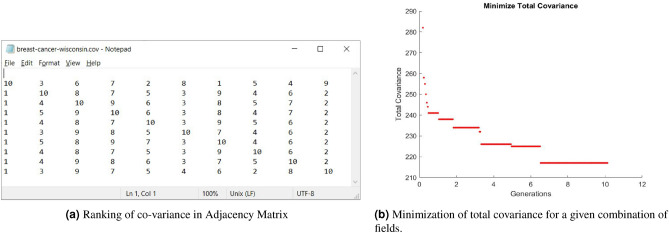
Minimization of covariance for WBC dataset.

As discussed in Section “Proposed methods”, different order of bar graphs for Type-1 and Type-3 transformations produce different images. A bar represents its corresponding field value of a given sample. We have tried to bring the related bars closer to each other by using a covariance matrix that determines the “closeness” of two fields. For example Fig. 6a shows the Adjacency Matrix of co-variance of each field for WBC dataset. The data is arranged row-wise such that each value represents the rank of *i*th row with *j*th column of a given field. To get the “best” arrangement of fields, we minimize the total co-variance rank by using a meta-heuristic algorithm GA to solve this shortest path problem. The process of minimization for WDBC is shown in Fig. 6b where the minimum rank is obtained by the end of 10th generation. The dataset fields were reorganized where the related fields were put close to each other according to the order of their similarity. The final order of fields for WBC and WDBC produced through minimum ranks are shown in Table 9. The images of these datasets were generated accordingly for the experiment. Notably, this order of fields does not have significant improvement over the original arrangement as the CNN produces similar convolved images.

**Table 9:** Order of fields based on minimization of total co-variance of adjacency matrix.

Dataset	Order of fields
WBC	[5, 4, 6, 2, 3, 7, 9, 1, 10, 8]
WDBC	[5, 27, 14, 16, 4, 11, 2, 10, 3, 6, 1, 7, 13, 29, 20, 24, 8, 21, 22, 17, 25, 26, 12, 30, 9, 18, 23, 19, 28, 15, 31]

The only shortcoming of the CNN algorithm is its high processing cost than other methods, especially with bigger sized images. Generally, it takes 9–15 s for a MATLAB 2018 program to complete the training process on DELL XPS i7-9700 @ 3GHz machine with 8 CPUs and NVIDIA GEFORCE RTX 2060 GPU. Despite this, the experimental results demonstrate the size of data has no direct impact on the performance of CNN. Additionally, with the advent of quantum computing^56^ and parallel GPUs with enough memory can produce results in a reasonable time frame. The data wrangling process of converting non-image data to the image is not too expensive either. The every-case time complexity of the bar graph approach has the order of *O*(*Nd*) and the normalized distance matrix has the order of $O(Nd^2)$. The Matlab code and data is available at https://github.com/anuraganands.

## Conclusion

The objective of this paper was to process non-image data (in a non-time series form) of Breast Cancer datasets WDBC and WBC into CNN due to its state-of-the-art performance and elimination of manual feature extraction for image recognition applications. The utilization of CNN has been confined largely to image data only except for some domain-specific data conversion techniques such as NLP and voice recognition. We have proposed three novel approaches to convert numerical non-time series data to image data. This process of conversion is very straightforward with the efficiency of the order of not more than $O(Nd^2)$. The experimental results on classification accuracy show the competitiveness of these methods. There is also a high potential for improving these approaches further to have more outstanding results. For example, bar graphs with different shapes, sizes, color and even arrangements can be tried. Similarly, distance matrix can be enhanced to have more information such as the mean/variance of the neighboring elements. It still needs to be seen how other applications with various types and orientations of numerical data would respond to CNN after non-image data conversion to image data. Intuitively, the more the information on data would produce the better the results as observed with the combined approach. Moreover, the imminent future work is to try our methods on time-series data to have competitive results with its counterpart of 1-D transformation. Finally, the classification accuracy of numerical data without any sophisticated data transformation on 1-D CNN did not produce acceptable results.

## Data Availability

The datasets analysed during the current study are available in the UCI repository, [https://archive.ics.uci.edu/ml/datasets/breast+cancer+wisconsin+(diagnostic) and https://archive.ics.uci.edu/ml/datasets/breast+cancer+wisconsin+(original)].
